# The role of PTEN in puromycin aminonucleoside-induced podocyte injury

**DOI:** 10.7150/ijms.72988

**Published:** 2022-08-15

**Authors:** Qi Ren, Shengyou Yu, Huasong Zeng, Huimin Xia

**Affiliations:** 1 The Second School of Clinical Medicine, Southern Medical University, Guangzhou, Guangdong, P.R. China.; 2 Department of Pediatrics, Guangzhou First People's Hospital, South China University of Technology, Guangzhou, Guangdong, P.R.China.; 3 Guangzhou Women and Children's Medical Center, Guangzhou, Guangdong, P.R. China.

**Keywords:** Podocyte, PTEN, Proteinuria, Autophagy, Apoptosis

## Abstract

Podocytes are specialized cells of the glomerulus that play important structural and functional roles in maintaining the filtration barrier. Loss and injury of podocytes are leading factors of glomerular disease and kidney failure. Recent studies found that phosphatase and tensin homolog (PTEN) may play a critical role in maintaining the normal structure and function in podocytes. However, we still understand very little about how PTEN is regulated under podocyte injury conditions. In this study, We therefore investigated whether PTEN could play a role in podocyte injury induced by puromycin aminonucleoside (PAN), and whether dexamethasone (DEX) alleviates podocyte injury by PTEN/PI3K/Akt signaling. Our results showed that PI3K/Akt pathway was activated in podocytes exposed to PAN conditions, accompanied by down-regulation of the PTEN and microtubule-associated light chain 3 (LC3) expression.podocyte-specific knockout of PTEN significantly promoted podocyte injury, The potential renoprotection of overexpressed PTEN in podocytes was partly attributed with an improvement in autophagy and the inhibition of apoptosis.These novel findings also suggest that targeting PTEN might be a novel and promising therapeutic strategy against podocyte injury.

## Introduction

Recent studies have found that podocytes are highly differentiated cells that represent a crucial mechanical and electrostatic barrier to prevent the generation of proteinuria 1-3. Although podocyte injury is the main cause of proteinuria, the underlying mechanism has not yet been elucidated. Phosphatase and tensin homolog deleted on chromosome 10 (PTEN) is a dual-specificity lipid and protein phosphatase that inhibits cell growth, cytoskeletal remodeling, and phagocytosis 4. One of the typical functions of PTEN is the inhibition of the phosphatidylinositol 3-kinase (PI3K)-AKT pathway 5-7. Therefore, some studies refer this pathway to PTEN/PI3K/Akt-signaling pathway. However, there has been no report on the mechanism by which the PTEN/PI3K/Akt-signaling pathway is involved in podocyte injury. A previous study has found that the PI3K/Akt signal transduction pathway plays an important role in podocyte injury 8. These results raise the possibility that PTEN may govern podocyte injury via the PI3K/Akt signaling pathway. Therefore, we speculate that PTEN maintains the structural and functional integrity of podocytes by stabilizing the PI3K/Akt signaling pathway, thereby inhibiting podocyte injury. In this study, our findings have proved the role of the PTEN/PI3K/Akt-signaling pathway in the regulation of podocyte injury. Our findings shed light on the pathogenesis of proteinuria and provide the theoretical basis for the molecular mechanism of podocyte injury.

## Materials and methods

### Podocyte culture and siRNA/adenovirus infection

Immortalized mouse podocytes were cultured as previously described 9. Briefly, the podocytes were cultured in 10% Roswell Park Memorial Institute medium 1640 (RPMI 1640, Gibco) at 33°C for proliferation. Podocytes were cultured at 37°C without interferon-gamma for 14 days to induce differentiation. To knock down PTEN, differentiated podocytes were transfected with Lipofectamine 2000 transfection reagent (Life Technologies, USA). The adenovirus (Biowit Technologies, ShenZhen, China) infected podocytes were performed for PTEN overexpress. Adenovirus generation, amplification, and titer were performed according topreviously described procedures 10-11, Infection with adenoviruses was performedata multiplicity of infection (MOI) of 100. Briefly, the podocytes were transfected with 2.0 mg vector DNA with 10ml Lipofectamine2000 in 2 ml serum-free Dulbecco's modified Eagle's medium (DMEM, Gibco) medium. At 6 h after transfection, the medium was replaced with normal DMEM/F12 medium with 10% foetal bovine serum (FBS,Gibco) for 24 h. To knock down PTEN, gene silencing of podocytes with PTEN was performed with Lipofectamine 2000 according to the manufacturers protocol, differentiated podocytes were transfected with 6 μl of PTEN siRNA mixed with 100 μl of siRNA transfection media (LifeTechnologies) for 30 min at room temperature.Subsequently, transfected podocytes were incubated for 48 h at 37℃.The sequences of three PTEN targeting siRNAs were as follows: PTEN siRNA-1, AGCTAAAGGTGAAGATATA; PTEN siRNA-2, AGTAAGGACCAGAGACAAA; PTEN siRNA-3, AGAAAGACTTGAAGGTGTA. The PCR primers used for real time PCR were PTEN-forward: CCAGTCAGAGGCGCTATGTAT. PTEN-reverse: GGCAGACCACAAACTGAGGAT.GAPDH-forward: AACAGCCTCAAGATCATCAGCA. GAPDH-reverse: ATGAGTCCTTCCACGATACCA. The relative expression levels of PTEN mRNA were determined bythe Ct(2^-ΔΔCt^) method. GAPDH was used for normalization.PTEN protein levelwas confired by immunoblot.

### Apoptosis Analysis

Podocytes exposed to growth restrictive conditions for > 14 days were allowed to attach to tissue culture plates for 24 h. Cells were then incubated with medium containing 10% fetal bovine serum in the presence or absence of DEX (Sigma, Co.). PAN was added to the medium after 1 h. The percentage of apoptotic elements was assessed for protein expression among cells harvested at 8 h, 24 h, and 48 h. Apoptosis was measured by staining 500 µL of cell suspension with fluorescein isothiocyanate (FITC)-conjugated Annexin V (Annexin V-FITC) (5 µL) and propidium iodide (PI) (5 µL) (Sigma, Co.) at 4°C for 10 min avoiding light. The apoptosis rates were examined by Fluorescence-activated cell sorting (FACS) Calibur flow cytometer (Becton Dickinson, USA).

### Immunofluorescence Analysis

The expression of PTEN in podocytes was detected using immunofluorescence, as described in a previous study 9. Briefly, the coverslips were incubated with anti-PTEN antibody (Abcam), and then incubated with goat anti-rabbit IgG horseradish peroxidase-conjugated secondary antibody for 1 h at room temperature (Life Technologies). Finally, coverslips were mounted and images were taken using an immunofluorescence microscope (Zeiss, Germany). We counted at least 200 nuclei in triplicate in each experiment.

### Western blotting Analysis

Western blotting was performed to measure the protein levels that were exposed to PAN in the presence and absence of DEX. Podocytes were harvested by trypsin digestion at 37°C for 3 min. Proteins were extracted for western blotting, which was performed using standard procedures. The protein concentration was determined by the bicinchoninic acid (BCA) protein assay kit (Pierce, Rockford, IL) according to the manufacturer's protocol. The blots were finally visualized using an enhanced chemiluminescence detection system (Tanon-5200Muilti, China). Quantitative analysis was performed using ImageJ Software.

### Transmission electron microscopy

Podocytes were prepared for transmission electron microscopy (TEM) (JEOL, Ltd., Tokyo, Japan). Ten cytoplasmic fields per grid were randomly captured per cell. The taken images from TEM were imported into Image-Pro Plus version 6.2 software (Media Cybernetics Inc) for morphometric area measurements of podocytes.

### Statistical Analysis

Statistical analysis was performed by means of IBM SPSS 22 (IBM Corp., Armonk, NY, USA). and GraphPad Prism version 7.0 was used for the figures. Normally distributed data were represented by means±standard deviation (SD). Independent samples t-tests were used to compare differences between groups. Data among multiple groups were analyzed with one-way analysis of variance (ANOVA) followed by Tukey's multiple comparisons test, Differences with p < 0.05 were considered to be statistically significant.Each experiment was repeated independently at least three times with similar results.

## Results

### Morphological observation of podocytes

**Figure 1:**
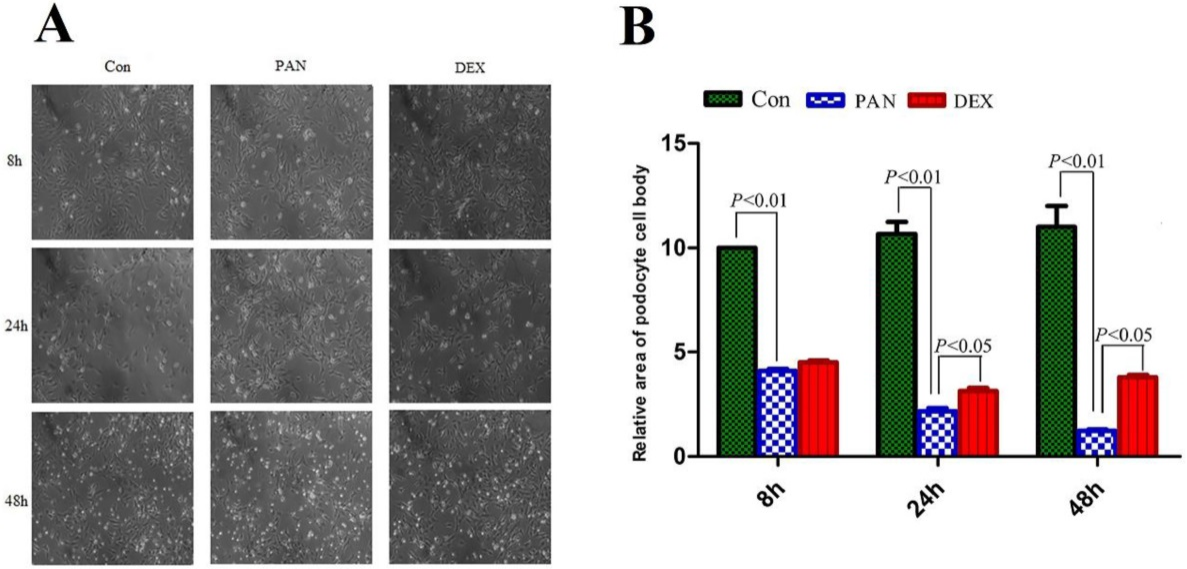
** Morphological changes of podocytes at different times for different groups.** (A) Representative pictures of podocyte in each group as indicated. (B) the relative area of podocytes in each group as indicated. foot processes and the connection between podocytes are intact in the control group. Foot processes appeared retracted, and the area of PAN-inducted podocytes was significantly reduced at 24 h. Moreover, the foot process retracted and disappeared at 48 h, and the connections between the cells disappeared. However, 24 and 48 h after DEX treatment, the foot processes and the connection between podocytes was still preserved. Data were presented as mean ± SD.

Podocytes were observed and photographed under an inverted microscope (Axiovert 25, German ZEISS). the podocyte cell area was manually traced along the cell perimeter using Image J software, and the relative area of podocytes was calculated by SPSS 19.0. The cell bodies and nuclei of PAN-induced podocytes significantly decreased, and the area of PAN-inducted podocytes was significantly reduced at 8 h (P < 0.01). Parts of the foot processes were lost at 48 h (P < 0.01). However, after DEX treatment, the area of podocytes was significantly greater at different time points (P < 0.05) (Fig. 1).

### Assessment of podocyte apoptosis

**Figure 2:**
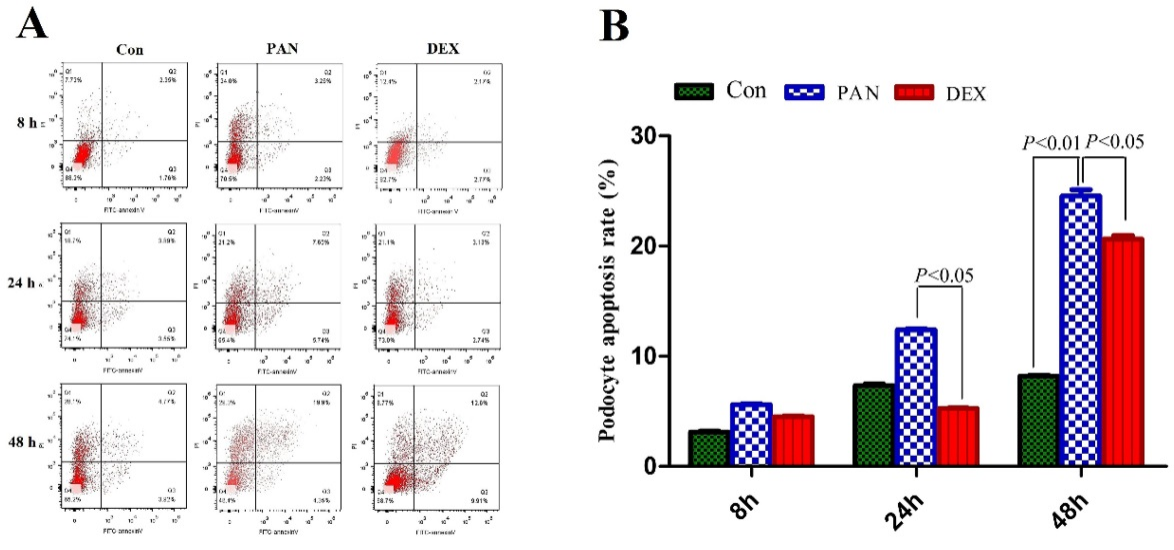
** Analysis of apoptosis was carried out by flow cytometry at the indicated time points.** (A) Representative podocyte apoptosis from each group as indicated. (B) The percentage of podocyte apoptosis in each group is indicated in the diagram as mean ± SD from three independent experiments.The percentage of apoptotic cells increased after 24 h in cells that were exposed to PAN without DEX, whereas cells that were treated with DEX were resistant to PAN-induced apoptosis. P < 0.05 versus PAN.

We first tested the hypothesis that DEX exerted an inhibitory effect on PAN-induced podocyte injury. To this end, we measured the number of viable cells among cultured immortalized podocytes grown under restrictive conditions for 12 days and then injured by exposure to PAN. We first measured the percentage of apoptotic cells. PAN-treated podocytes showed greatly enhanced apoptosis 8 h after treatment compared to control. Apoptosis was even higher after 24 h of PAN treatment, and most podocytes were apoptotic by 48 h. The DEX group showed significantly lower rates of apoptosis than the PAN-treated group at all time points. Therefore, DEX significantly decreased PAN-induced podocyte apoptosis (P < 0.05 vs. control). As shown in Fig. 2, these results supported the notion that DEX exerts anti-apoptotic effects on cells exposed to PAN.

### Effect of DEX on PAN-induced PTEN distribution changes in podocytes

**Figure 3:**
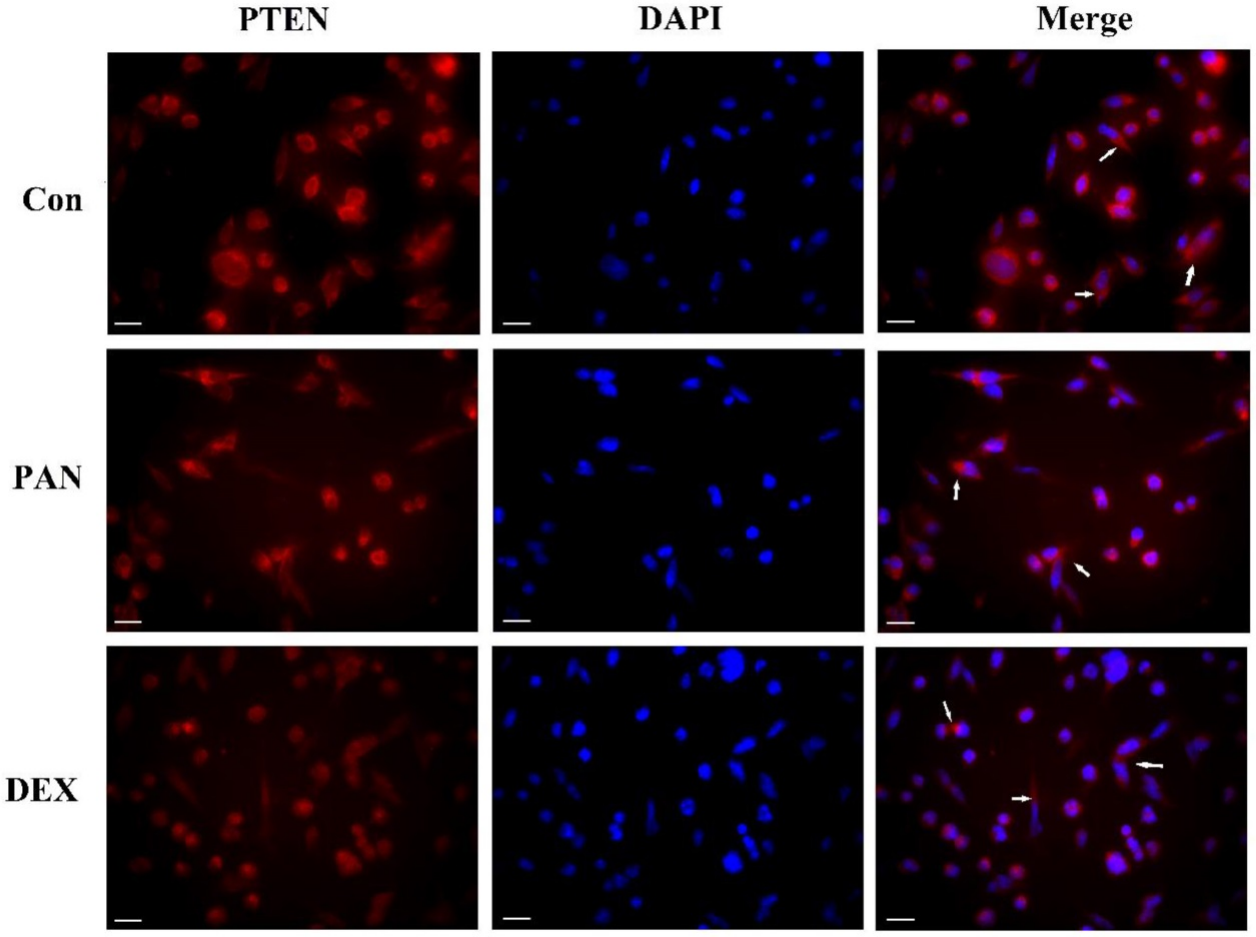
** Representative pictures of anti-PTEN immunofluorescence.** Immunofluorescence analysis of PTEN puncta in podocytes of each group as indicated (white arrows), PTEN expression was weak in normal podocytes, while PAN-injured podocytes had decreased PTEN expression compared to the control group (P < 0.05). After the pretreatment of podocytes by DEX, PTEN expression increased significantly compared to the PAN group (P < 0.05). The white scale bar indicated 20 μm.

To investigate the role of PTEN in podocyte injury, we first determined the expression of PTEN in the podocytes treated with PAN and control. The PAN-injured podocytes showed decreased PTEN expression compared to the control group. In contrast, after DEX treatment, PTEN, which significantly improved, was more homogeneously distributed in the plasma membrane. Our data indicated that PTEN contributed to podocyte injury, as evidenced by its downregulated expression in podocytes (Fig. 3).

### Effect of DEX on PAN-induced PTEN protein expression

**Figure 4:**
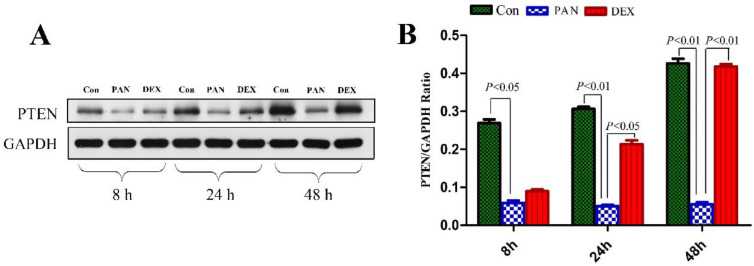
** Western blot analysis of PTEN in podocytes from the different groups.** (A) Representative pictures of PTEN protein expression in the podocytes from each group as indicated. (B)Bar graphs indicated relative levels of PTEN normalized to GAPDH.

To elucidate the mechanisms of PAN-induced podocyte injury and the potential effects of DEX on specific pathways, we performed western blotting to measure the protein expression of PTEN. Western blotting showed that PTEN expression was significantly decreased in the PAN group (P < 0.01) compared to the control. In contrast, DEX completely prevented the PAN-induced decrease in PTEN. These results demonstrated that the incubation of cultured podocytes with PAN decreased PTEN protein levels, which was prevented by the addition of DEX (P < 0.05 versus PAN) (Fig. 4).

### Effect of DEX on PAN-Induced PI3K p85 Protein Expression

**Figure 5:**
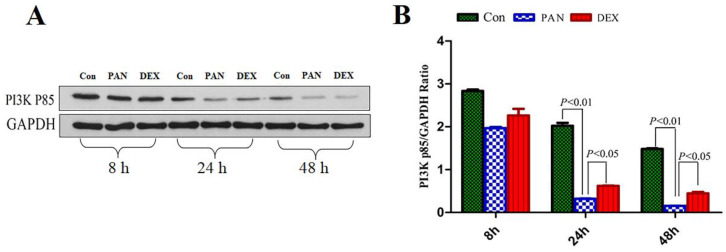
** Western Blot analysis of PI3K p85 and GAPDH at different time points.** (A) Representative pictures of PI3K p85 protein expression in the podocytes from each group as indicated. (B)Bar graphs indicated relative levels of PI3K p85 normalized to GAPDH.

To elucidate the mechanisms of PAN-induced podocyte injury and the potential effects of DEX on the PTEN/PI3K/Akt signaling pathway, we performed Western blot analysis to measure the protein expression of PI3K p85, Our results showed that the protein expression of the PI3K p85 in the PAN group was lower than that in the control at 8 h and showed a downward trend over time. PI3K p85 protein expression continued to decrease at 24 h and 48 h (p<0.01). After DEX treatment,the protein expression of the PI3K p85 was not significantly different from that in the PAN group at 8 h, However it was higher than that in the PAN group at 24 h and 48 h (p<0.05) (Fig. 5).

### Effect of DEX on PAN-Induced p-Akt Protein Expression

**Figure 6:**
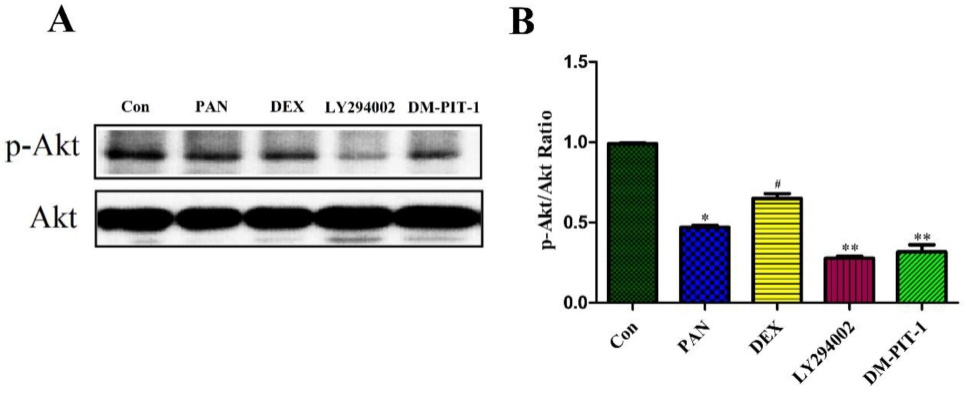
** Western Blot analysis of p-Akt and Akt in the podocytes from the different groups.** (A) Representative pictures of p-Akt protein expression in the podocytes from each group as indicated. (B)Bar graphs indicated relative levels of p-Akt normalized to Akt. *p<0.05 vs control,^#^p<0.05 vs PAN group, **p<0.01 vs DEX group.

To elucidate the mechanisms of PAN-induced podocyte injury and the potential effects of DEX on the PTEN/PI3K/Akt signaling pathway, we performed Western blot analysis to measure the protein expression of p-Akt, Taking total Akt as the internal reference, the protein expression of p-Akt decreased after podocyte injury induced by PAN, and its expression was significantly lower than in the control group (p<0.05). DEX treatment significantly inhibited the above mentioned effects of PAN. After DEX treatment, the expression of p-Akt was significantly higher than that in the PAN group, while LY294002 pretreatment for 1 h basically inhibited the phosphorylation level of Akt in the podocytes (p<0.01), In order to integrated different aspects to validate such conclusion. We performed to block the signaling pathway by using PIP3 Antagonist II, DM-PIT-1. The results demonstrated that the expression of p-Akt in the DM-PIT-1 group was significantly lower than that in the Control group (p<0.01) (Fig. 6).

### Effect of DEX on PAN-Induced p-GSK3β Expression

**Figure 7:**
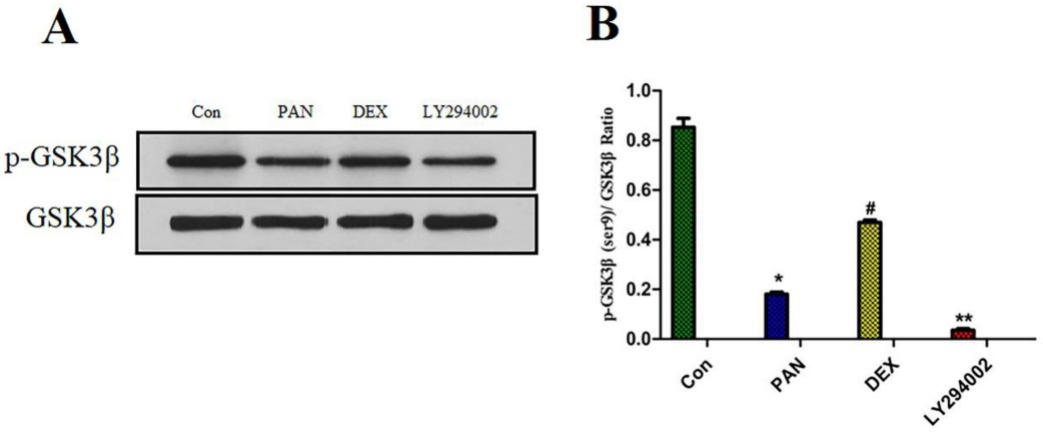
** Western Blot analysis of p-GSK3β and GSK3β in each experimental group.** (A) Representative pictures of p-GSK3β protein expression in the podocytes from each group as indicated. (B)Bar graphs indicated relative levels of p-GSK3β normalized to GSK3β. we found that PAN decreased the expression of p-GSK3β protein, The effect of PAN was reversed by DEX co-treatment,Expression of p-GSK3β was almost completely blocked by LY294002.*p<0.05 vs control,^#^p<0.05 vs PAN group, **p<0.01 vs DEX group.

To elucidate the mechanisms of PAN-induced podocyte injury and the potential effects of DEX on the PTEN/PI3K/Akt signaling pathway, we performed Western blot analysis to measure the protein expression of p-GSK3β. Taking total GSK3β as the internal reference, the protein expression of p-GSK3β was significantly reduced after podocyte injury induced by PAN; After DEX treatment, we found that the effects of PAN described above could be significantly inhibited. and the protein expression of p-GSK3β was significantly higher than in the PAN group, and the phosphorylation level of GSK3β in podocytes was basically suppressed after pretreatment with LY294002 (p<0.01) (Fig. 7).

### The protein expression of the autophagy markers LC3B

**Figure 8:**
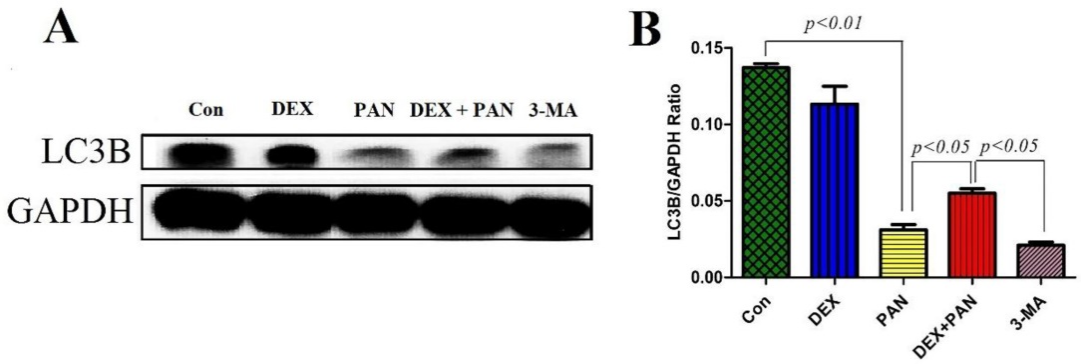
** Western blot analysis of LC3B in podocytes from the different groups.** (A) Western blot analysis of LC3B in the podocytes from each group as indicated. (B) Bar graphs indicated the relative levels of LC3B normalized to GAPDH.

To evaluate the role of autophagy on PAN-induced podocyte damage, western blot was performed to analyze the protein expressions in podocytes treated with PAN, DEX, and 3-MA (Autophagy inhibitor 3-methyladenine). The results showed that 3-MA and PAN could significantly inhibit podocyte autophagy, as evidenced by the decreased expression of LC3B protein (P < 0.05). After DEX treatment, autophagy was activated in podocytes, and the expression of LC3B significantly increased (Fig. 8).

### Expression of related proteins after silencing and overexpression of PTEN

**Figure 9:**
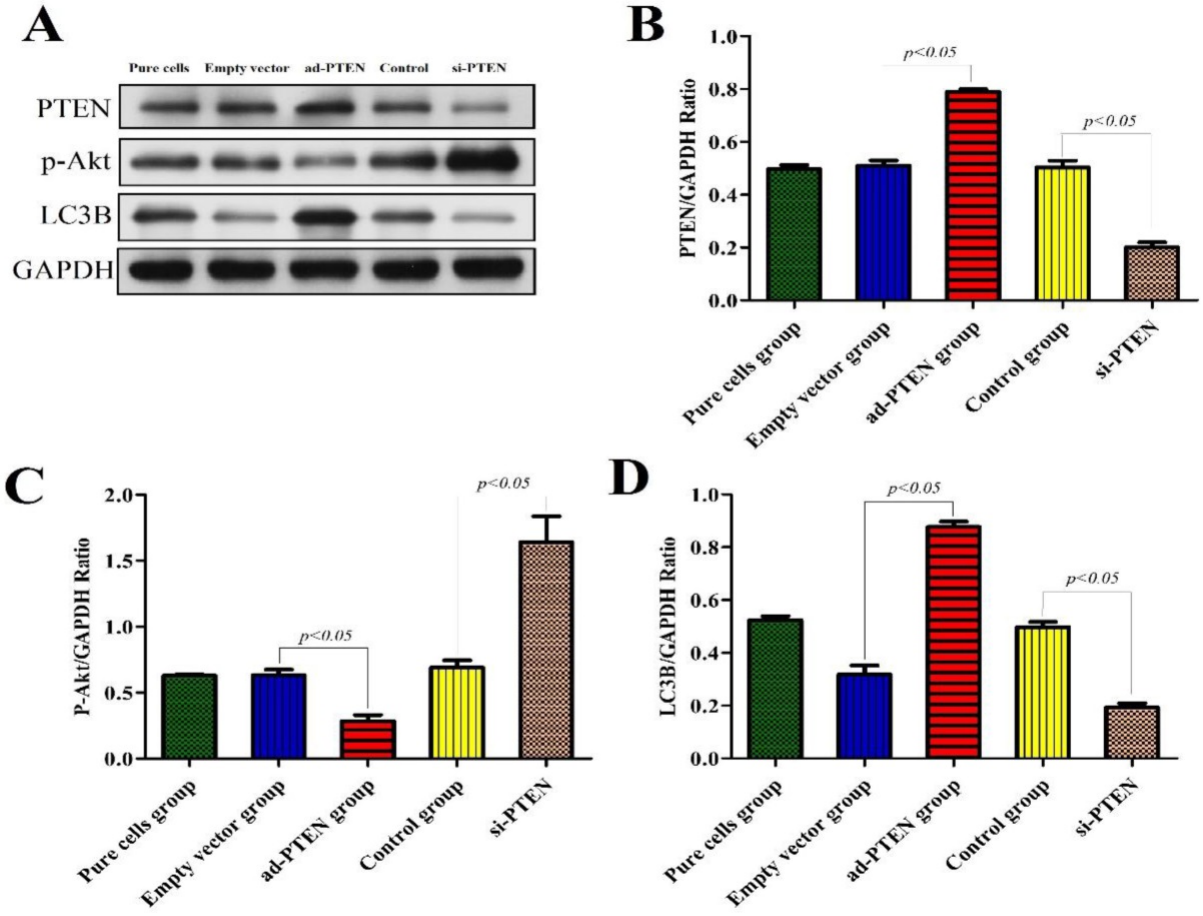
**Western blot analysis of PTEN, p-Akt and LC3B in podocytes from the different groups.** (A)Western blot analysis of PTEN, p-Akt and LC3B in the podocytes from each group as indicated. (B-D)Bar graphs indicated the relative levels of PTEN, p-Akt and LC3B normalized to GAPDH.

To evaluate the regulatory effect of PTEN on the PI3K/AKT signaling pathway and its effect on autophagy, we constructed a podocyte model of PTEN gene silencing and overexpression and performed western blotting on the podocyte proteins of each group. The results showed that after PTEN gene silencing, the expression of p-Akt increased, podocyte autophagy was significantly inhibited, and the expression of the LC3B protein significantly decreased (P < 0.05). Following PTEN gene overexpression, the expression of p-Akt decreased, podocyte autophagy was activated, and the expression of LC3B significantly increased. These results suggested that the inhibition of PTEN suppressed autophagy and aggravated podocyte injury, and that elevation of PTEN restored autophagy and protected podocytes (Fig. 9).

### Assessment of podocyte injury by TEM

**Figure 10:**
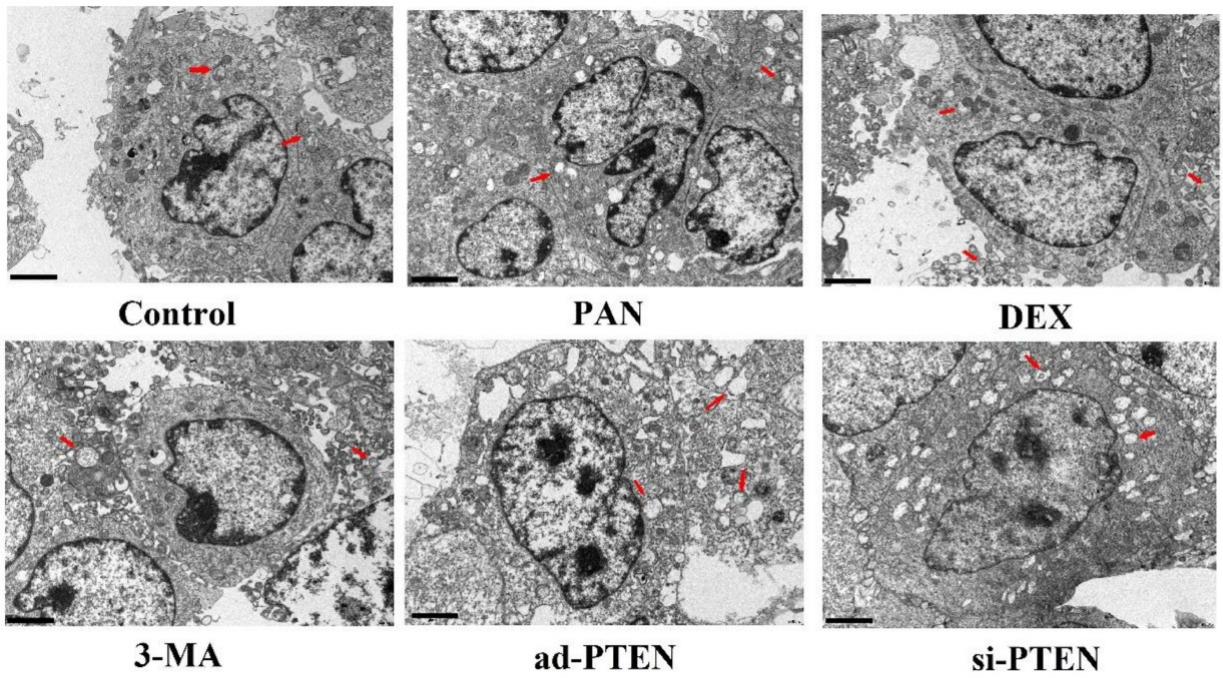
** Representative ultrastructural imagines showing autophagosomes in podocytes.** Autophagosomes (red arrows), In the control group, the membrane surface of the podocytes had protrusions, the nuclei were irregular, the endoplasmic reticulum structure was clear, and autophagosomes were visualized in the cytoplasm. Compared to the control group, the number of autophagosomes was markedly lower in the PAN-treated podocytes. Compared to the control group, the number of autophagosomes was markedly lower in the 3-MA-treated podocytes. Compared to the PAN group, the number of autophagosomes was markedly increased following DEX treatment.The black scale bar indicated 2 μm.

TEM was used to observe the effect of individual treatments on autophagy. The podocytes in the control group rarely contained isolated autophagosomes with double-layer or multilayer membranes. In the PAN group, the cytoplasm contained many vacuoles and few autophagosomes. The number of autophagosomes in the cytoplasm of podocytes increased following treatment with DEX. Compared to the control, the number of autophagosomes in the cytoplasm of podocytes in the si-PTEN group was significantly reduced. Compared to the PNA and 3-MA groups, the number of autophagosomes in the cytoplasm of podocytes in the ad-PTEN group was significantly increased (P < 0.05). These results suggested that the inhibition of PTEN suppressed autophagy and aggravated podocyte injury, and that elevation of PTEN restored autophagy and protected podocytes (Fig. 10).

## Discussion

Podocytes are an important structural and functional component of the glomerular filtration membrane and the final protective barrier against protein leakage. Podocyte impairment is the main reason for proteinuria. Recent studies have revealed that podocyte injury can be induced by PAN, angiotensin II (Ang II), cyclosporine A, and stretching. However, the mechanism underlying podocyte injury and the development of proteinuria has not yet been elucidated. Glucocorticoids are widely used to treat podocyte diseases and proteinuria, and many studies have suggested that podocytes contain functional glucocorticoid receptors; therefore, DEX may exert a direct effect on podocytes contributing to their survival. We reasoned that abnormal PTEN expression produces an imbalance in podocyte function, changing the filtration rate and causing proteinuria. DEX reduced podocyte injury by increasing the expression of PTEN, thus playing a protective role against the development of proteinuria.

The current study demonstrated that PTEN was downregulated following podocyte injury *in vitro*. Furthermore, the underlying renoprotection mechanism of PTEN was partly associated with the improvement of autophagy and motility, and the inhibition of apoptosis in podocyte injury. PTEN is a dual-specificity lipid and protein phosphatase that inhibits cell growth, cytoskeletal remodeling, and phagocytosis 4. As an upstream inhibitor of the PI3K/Akt signal transduction pathway, the most important substrate of PTEN is phosphatidylinositol (3, 4, 5)-triphosphate (PIP3). PIP3 is the product of phosphatidylinositol-3' kinase (PI3K) and mediates the activation of AKT. PTEN dephosphorylates PIP3 to maintain a low level of PIP3, thereby inhibiting the PI3K/AKT pathway. Therefore, some scholars refer to this pathway as the PTEN/PI3K/AKT signal pathway. Several studies have shown that PTEN is involved in the progression of kidney disease 12-14. Moreover, in animal models of diabetic nephropathy, the expression of PTEN in glomerular mesangial cells and podocytes is significantly downregulated, suggesting that PTEN may play an important role in glomerular sclerosis. In addition, as the expression of PTEN in the kidney is downregulated, podocytes are damaged, and urine protein levels gradually increase 15-17, suggesting that PTEN may be a protective gene in the kidney. The PI3K/Akt signaling pathway is widely present in eukaryotic cells and participates in many physiological and pathological processes, such as cell growth, differentiation, and proliferation. Studies have shown that in some disease states, stabilizing the PI3K/Akt signaling pathway can effectively alleviate damage to podocytes and reduce the incidence of proteinuria 18-19. Recently, studies have found that podocytes possess functional receptors for glucocorticoids, and that glucocorticoids can act directly on podocytes to protect them 20-23. Glucocorticoids can mediate the activation of the PI3K/Akt pathway, inhibit cell apoptosis through a variety of mechanisms, and promote cell survival. Huber et al. showed that CD2AP can bind to PI3K p85 in podocytes and activate the PI3K/Akt signaling pathway 24-25. A previous study also found that DEX can reverse PAN-induced podocyte apoptosis by regulating the PI3K/Akt signaling pathway 8.

In this study, we found that PAN-induced podocyte injury decreased the expression of PTEN, increased the rate of podocyte apoptosis, and inhibited autophagy. The distribution of PTEN in podocytes was abnormal, and there was a loss of PTEN distribution in the cell membrane and cytoplasm, all of which were reverse by DEX treatment. Furthermore, the expression of LC3B proteins decreased after PAN-induced podocyte injury, which, again, was reversed by DEX treatment. Studies have also found that PTEN may be a protective gene in the kidney 26-30. Elevation of PTEN can negatively regulate the PI3K/Akt pathway, inhibit the activation of p-Akt, improve the phenotype of podocytes, and reduce podocyte injury 31. In early diabetic nephropathy, as glomerular damage increases, the expression of PTEN gradually decreases, suggesting that PTEN correlates with glomerular damage 32-35. To confirm the exact mechanism by which PTEN functions in podocyte injury, we silenced the PTEN gene. As a result, we found that the expression of p-AKT increased, podocyte autophagy was significantly inhibited, and the expression of the LC3B protein significantly decreased. Following overexpression of PTEN, the expression of p-AKT decreased, podocyte autophagy was activated, and the expression of LC3B significantly increased. Additionally, TEM showed that when PTEN was silenced, the mitochondria of podocytes gradually swelled and became rounded, the mitochondrial cristae arrangement was disordered, and autophagy was inhibited, but all of these were reversed by DEX treatment. This finding is consistent with other relevant studies worldwide.

In summary, this study has clarified the role and significance of PTEN in podocyte impairment and repair, shedding further light on the signaling mechanisms by which DEX inhibits PAN-induced podocyte injury. These novel findings also suggest that targeting PTEN might be a novel and promising therapeutic strategy against podocyte injury.
